# Knocking down CDK4 mediates the elevation of let-7c suppressing cell growth in nasopharyngeal carcinoma

**DOI:** 10.1186/1471-2407-14-274

**Published:** 2014-04-21

**Authors:** Zhen Liu, Xiaobin Long, Cheng Chao, Chen Yan, Qiangyun Wu, Shengni Hua, Yajie Zhang, Aibing Wu, Weiyi Fang

**Affiliations:** 1 Department of Pathology, Guangzhou Medical University, Guangzhou 510182, China; 2 Cancer Research Institute, Southern Medical University, Guangzhou 510515, China; 3 Cancer Center of Affiliated Hospital, Guangdong Medical College, Zhanjiang 524001 PR, China; 4 Otorhinolaryngology of Zhujiang Hospital, Southern Medical University, Guangzhou 510282 PR, China; 5 Pediatric Center of Zhujiang Hospital, Southern Medical University, Guangzhou 510282 PR, China; 6 Tumor Center of Integrated Chinese and Western medicine Hospital, Southern Medical University, Guangzhou 510315 PR, China

**Keywords:** NPC, CDK4, let-7c, Prognosis

## Abstract

**Background:**

CDK4 is a protein kinase in the CDK family important for G1/S phase cell cycle progression. However, the roles and molecular mechanisms of CDK4 triggering nasopharynx carcinogenesis are still unclear.

**Methods:**

Lentiviral-vector mediated shRNA was used to suppress CDK4 expression and examine its molecular mechanisms. Using immunohistochemistry, we analyzed CDK4 protein expression in clinicopathologically characterized nasopharyngeal carcinoma (NPC) cases and nasopharyngeal tissues (NPs). Survival curves were plotted by the Kaplan-Meier method and compared using the log-rank test.

**Results:**

In this investigation, we knocked down CDK4 expression and observed that NPC cell growth and cell cycle progression were significantly blocked by suppressing expression of CCND1, CDK6, and E2F1 as well as elevated p21 expression. Further, we found that reduced CDK4 expression elevated the expression of let-7c, a tumor-suppressive miRNA modulated by E2F1. We found that let-7c was markedly downregulated in NPC tissues compared to NPs and suppressed cell growth and cell cycle progression by modulating p15/p16/CDK4/E2F1 pathway. Finally, CDK4 protein was observed to be overexpressed in NPC tissues and could be considered an unfavorable prognosis factor for NPC patients although its independent prognostic value did not reach statistical significance (p = 0.087).

**Conclusions:**

Our results demonstrated that overexpressed CDK4 is an unfavorable prognostic factor which suppresses the expression of tumor suppressive-factor let-7c through p21/CCND1/CDK6/E2F1 signaling, and inhibits cell proliferation by p15/p16/CDK4/E2F1 feedback signaling in NPC.

## Background

NPC is one of the most common carcinomas in Southern China and exhibits a highly malignant phenotype. Clinically, NPC is classified as a specific type of head and neck squamous cell carcinoma with its unique epidemiology, clinical characteristics, etiology, and histopathology. Therefore, separate efforts are still needed to investigate its underlying molecular mechanisms of carcinogenesis. Synergetic effects of viral infections, genetic alterations, and environmental factors are key factors driving the aberrant activity of a variety of genes and signal pathways during NPC pathogenesis. Epstein-Barr virus-encoded LMP1 promotes proliferation and transformation of human nasopharyngeal epithelial cells by inhibiting LKB1-AMPK pathway [1]. A single nucleotide polymorphism -32G/A in the promoter region of LOC344967 gene creates an activator protein (AP-1)-binding site in its transcriptional regulatory region, which significantly enhanced the binding of AP-1 to the promoter region of LOC344967 and activated its expression in vivo [2]. Tobacco smoking as a risk factor for NPC has been supported by multiple studies [3,4]. Cigarette smoke extract promoted EBV replication, induced the expression of the immediate-early transcriptional activators Zta and Rta, and increased transcriptional expression levels of BFRF3 and gp350 in the lytic phase [3].

CDK4 is a member of the cyclin-dependent kinase family and highly similar to the gene products of *S. cerevisiae* cdc28 and *S. pombe* cdc2. It is a catalytic subunit of the protein kinase complex including CDK4, CDK6, and CCND1 important for G1 to S cell cycle progression. CDK4 was observed to have higher oncogenic activity than oncogenic transcript factor CCND1 and it markedly enhanced malignant skin tumorigenesis in CDK4 transgenic mice [5]. Furthermore, overexpression of CDK4 has been observed in many tumor types, including oral squamous cell carcinoma [6], pancreatic endocrine tumors [7], lung cancer [8,9], and nasopharyngeal carcinoma [10], suggesting that CDK4 is a significant factor in promoting the initiation and development of tumors. However, the role of CDK4 and its mediated miRNA expression in the pathogenesis of NPC have not been reported. In this study, we found that knocking down CDK4 expression elevated the expression of tumor suppressor let-7c by modulating the G1/S transition cell signaling pathway, which in turn suppressed cell growth by through the p15/p16/CDK4/E2F1 pathway. Furthermore, overexpression of CDK4 was considered an unfavorable factor associated with NPC progression and poor prognosis.

## Methods

### Sample collection and cell culture

Cell culture and sample collection. Two NPC cell lines 5-8F and 6-10B were obtained from Cancer Research Institute of Southern Medical University and maintained in RPMI 1640 medium supplemented with 10% newborn calf serum (NBCS) (PAA Laboratories, Inc, Pasching, Austria) in a humidified chamber with 5% CO_2_ at 37°C. 133 paraffin-embedded undifferentiated NPC specimens with clinical prognosis information and 34 paraffin-embedded nasopharynx specimens (Table 1) were obtained at the time of diagnosis before any therapy from People’s Hospital in Zhongshan City (Guangdong, China). Among 133 patients, there were 17 patients treated by radiotherapy alone, 3 by chemocherapy, 105 by combine radiotherapy and chemocherapy. However, there were 8 patients who did not accept any treatment. All 56 fresh NPC and 15 NP samples (13 cases for chronic nasopharyngitis tissuses and 2 cases for normal nasopharyngeal tissues) were obtained from an otorhinolaryngologist using nasal endoscope. Subsequently, all samples were immediately stored in liquid nitrogen. The clinical processes were approved by the Ethics Committees of People’s Hospital of Zhongshan City and patients gave informed written consent. The pathologic stage of all specimens was confirmed according to the 1997 NPC staging system of the UICC.

**Table 1:** CDK4 is highly expressed in NPC tissues compared to NPs

**Protein expression (n)**			
			**P Value**
Expression level	NPs	NPCs	P = 0.001
Low	26	58	
High	8	75	
Total	34	133	

### RNA isolation, reverse transcription, and qRT-PCR

RNA was extracted from the NPC cell lines, NPC tissues and normal nasopharynx tissues using Trizol (Takara, Shiga, Japan). For miR-let-7c qRT-PCR expression analysis, mature miRNAs were reverse-transcribed, and real-time PCR was performed using All-in-One™ miRNA qRT-PCR Detection Kit following the manufacturer’s protocol. (GeneCopoeia™, Cat.No: AOMD-Q020). All data were normalized to U6 expression. Assays were performed in accordance with manufacturer’s instructions (Takara, Shiga, Japan). PCR reactions for each gene were repeated three times. miRNA expression was normalized to U6 according to the following calculations: 1) First, calculating Delta C(T) value of each sample (Targeted gene Ct value-Housekeeping gene Ct value); 2) Second, calculating -Delta Delta C(T) value of each sample (each sample Delta C(T) value -the maximal Delta C(T) value of all sample Delta C(T) values; 3) Third, the expression level of each sample was transformed to fold-relative value including that sample with maximal Delta C(T) value by 2(-Delta Delta C(T)) method [11]. 4) Finally, the differential expression level was analyzed between objective group and control group by *t* test.

### Immunohistochemistry and evaluation of staining

Immunohistochemistry and evaluation of staining of CDK4 (Santa Cruz Biotechnology, Santa Cruz, USA) were performed in NPC and NP tissues according to the previous description [12].

### Western blot analysis

Western blot was carried out according to the previous description [13,14] with rabbit polyclonal anti-CDK4 antibody, anti-ACTB, p21, E2F1, C-Myc antibody (1:400; Santa Cruz Biotechnology, Santa Cruz, USA); p15 and p16 antibody (Cell signaling technology, Danvers, USA), CCND1 antibody (1:500; Epitomics, Burlingame, USA). An HRP-conjugated anti-rabbit IgG antibody was used as the secondary antibody (Zhongshan, Beijing, China). Signals were detected using enhanced chemiluminescence reagents (Pierce, Rockford, IL).

### Establishment of NPC 5-8F cell line with stable expression of CDK4 short hairpin RNA

The preparation of lentivirus expressing human CDK4 short hairpin RNA (shRNA-509,1097) was reported by us using the pLVTHM-GFP lentiviral RNAi expression system [12]. NPC 5-8F cells were infected with lentiviral particles containing specific or negative control vectors, and polyclonal cells with GFP signal were selected for further experiments using FACS flow cytometery.

### Transient transfection with let-7c mimics and its inhibitor

Let-7c and its inhibitor were designed and synthesized by Guangzhou RiboBio (RiboBio Inc, China). Twenty-four hours prior to transfection, NPC cells 6-10B and 5-8F were plated onto a 6-well plate or a 96-well plate (Nest, Biotech, China) at 30–50% confluence. They were then transfected into cells using TurboFectTM siRNA Transfection Reagent (Fermentas, Vilnius, Lithuania) according to the manufacturer's protocol. Cells were collected after 48-72 hr for further experiments.

### Cell proliferation analysis

Cell proliferation was analyzed using MTT assay as described previously [13]. For shRNA-CDK4, the cells were incubated for 1, 2, 3, 4, 5, 6, or 7 d. For let-7c mimics or its inhibitor, the cells were incubated for 1, 2, or 3d.

### Colony formation assay

Cells were plated in 6-well culture plates at 100 cells/well. Each cell group had 2 wells. After incubation for 12 days at 37°C, cells were washed twice with PBS and stained with Giemsa solution. The number of colonies containing ≥ 50 cells was counted under a microscope. The colony formation efficiency was calculated as (number of colonies/number of cells inoculated) × 100%.

### Cell cycle assay

To evaluate cell cycle distributions, cells were fixed in 70% ice-cold ethanol for 48 hours at 4°C, and stained by incubating cells with PBS containing 10 μg/mL propidium iodide and 0.5 mg/mL RNase A for 15 min at 37°C, and analyzed for the DNA content of labeled cells by FACS Caliber Cytometry (BD Bioscience, USA). Each experiment was done in triplicate.

### Statistical analysis

All data were analyzed for statistical significance using SPSS 13.0 software. The Chi-square test was applied to the examination of the differences of CDK4 expression between normal epithelium and cancer tissues of nasopharynx as well as the relationship between CDK4 expression levels and clinicopathologic characteristics. Survival analysis was performed using Kaplan-Meier method. Two-tailed Student's *t* test was used for comparisons of two independent groups. One-way ANOVA was used to determine the differences between groups for all in vitro analyses. A P value of less than 0.05 was considered statistically significant.

## Results

### Stably downregulated CDK4 expression suppresses cell proliferation and cell cycle progression *in vitro* in NPC

**Figure 1:**
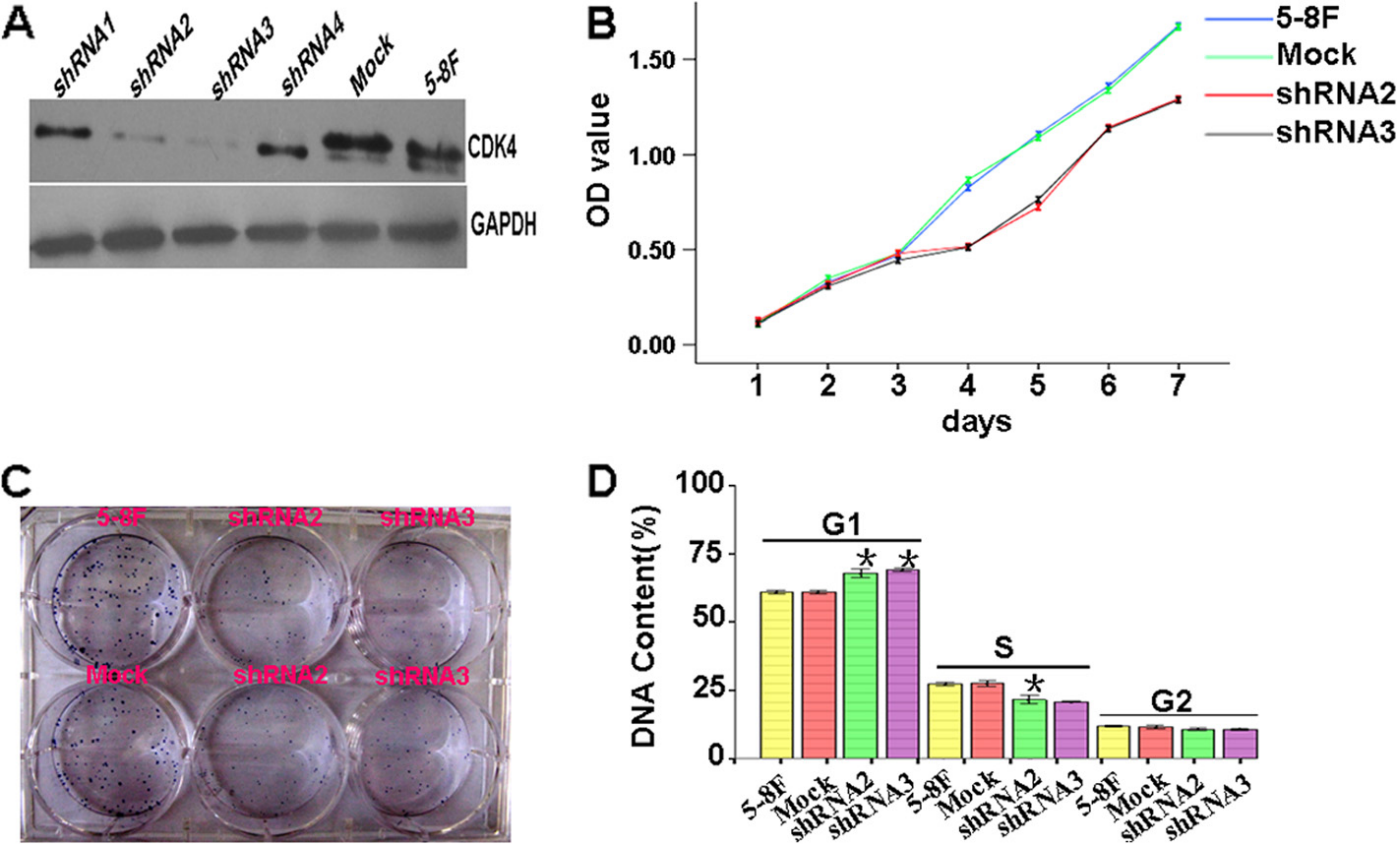
**Stably knocking down CDK4 expression suppresses cell proliferation and cell cycle progression in vitro in NPC. A**. CDK4 protein expression was stably suppressed by lentivirus-mediated shRNA in NPC cells. **B**. In vitro viability of NPC cells was decreased in CDK4-suppressed cells compared to PLV-Ctr cells by MTT assay. **C**. *In vitro* proliferative ability of NPC cells was significantly decreased in CDK4-suppressed cells compared to PLV-Ctr cells by colony formation assay. **D**. Stably downregulated CDK4 expression blocked cell cycle transition from G1 to S in shRNA-CDK4-2 and 3 cells. Data are presented as mean ± SD for three independent experiments (*p < 0.05).

In previous study, we demonstrated that suppressing CDK4 expression using lentiviral-mediated shRNA inhibited cell proliferation and G1 to S cell cycle transition. In this study, we used lentiviral-mediated shRNA specifically targeting CDK4 to stably inhibit expression of CDK4 in the 5-8F cell line. Two single clone cells shRNA2 and shRNA3 with stable suppression of CDK4 protein compared to mock vector and 5-8F cell lines were identified (Figure 1A). Subsequently, we observed that cell proliferation ability was significantly reduced in comparison to Mock cells by MTT (Figure 1B) and plated clone analysis (Figure 1C). Further, we found that knocking down CDK4 expression delayed the transition of cell cycle from G1 to S phase. These results suggested a significant proliferative effect of CDK4 on tumorigenesis in vitro (Figure 1D).

### Knocking down CDK4 elevates the expression of let-7c by modulating G1/S cell cycle signal in NPC

**Figure 2:**
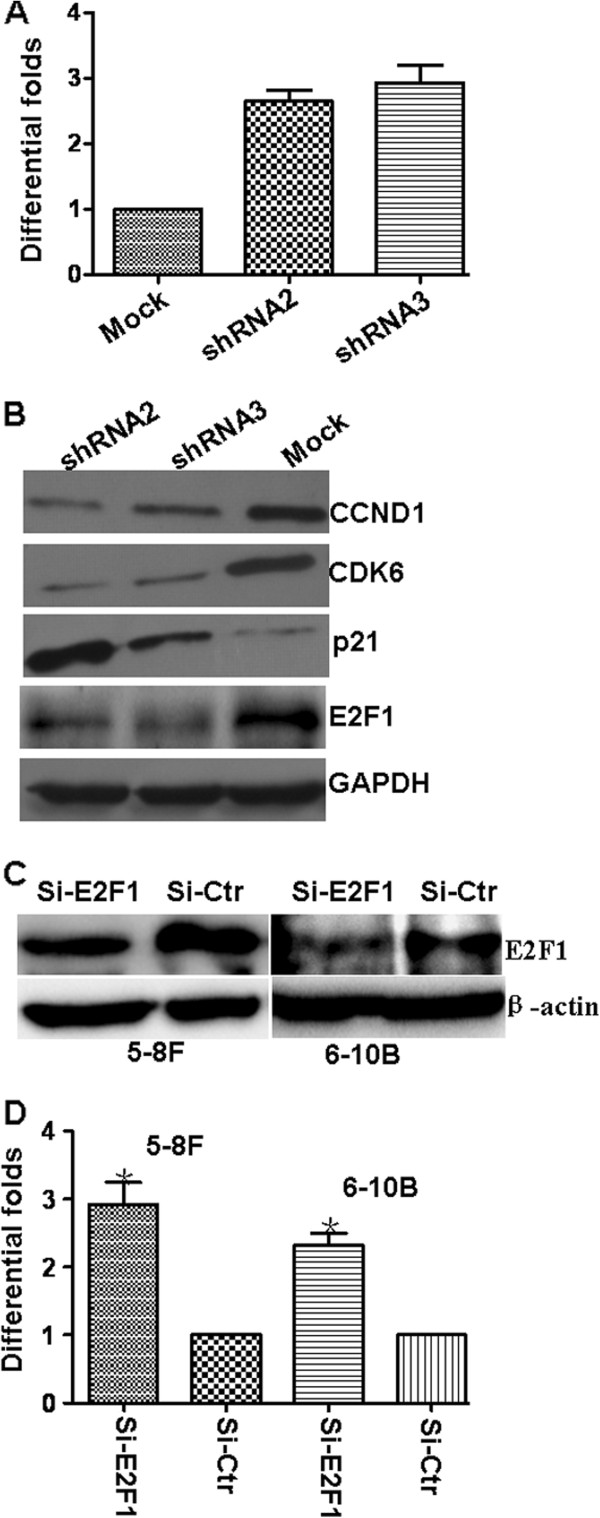
**Repression of CDK4 elevates expression of let-7c by modulating G1/S cell cycle signaling in NPC. A**. Let-7c expression was significantly increased in NPC cells after stable knockown of CDK4 expression. **B**. Knocking down the expression of CDK4 decreased the CCND1, CDK6, and E2F1 as well as activated p21 expression. **C**. E2F1 expression was markedly suppressed using specific siRNA-E2F1. **D**. Knocking down E2F1 by specific siRNA-E2F1 elevated the expression of let-7c.

Let-7c is a tumor-suppressive miRNA identified in some tumors [15,16]. In this study, we used qPCR to examine let-7c expression after knocking down CDK4 NPC cells. Interestingly, let-7c expression was markedly upregulated after suppressing CDK4 expression (Figure 2A). CDK4 is not a transcription factor and it was unlikely to directly control the expression of let-7c. However, based on predicted binding sites of E2F1 in let-7c promoter, we suspected that reduced CDK4 mediated the elevation of let-7c through suppression of E2F1 cell cycle signaling. We observed that knocking down expression of CDK4 decreased expression of CCND1, CDK6, and E2F1 yet upregulated p21 (Figure 2B). Suppressing E2F1 expression by siRNA stimulated let-7c expression in NPC 5-8F and 6-10B cells (Figure 2C,D). Our results revealed that knocking down CDK4 elevated the expression of let-7c by modulating G1/S cell cycle signaling in NPC.

### Let-7c is downregulated in NPC

**Figure 3:**
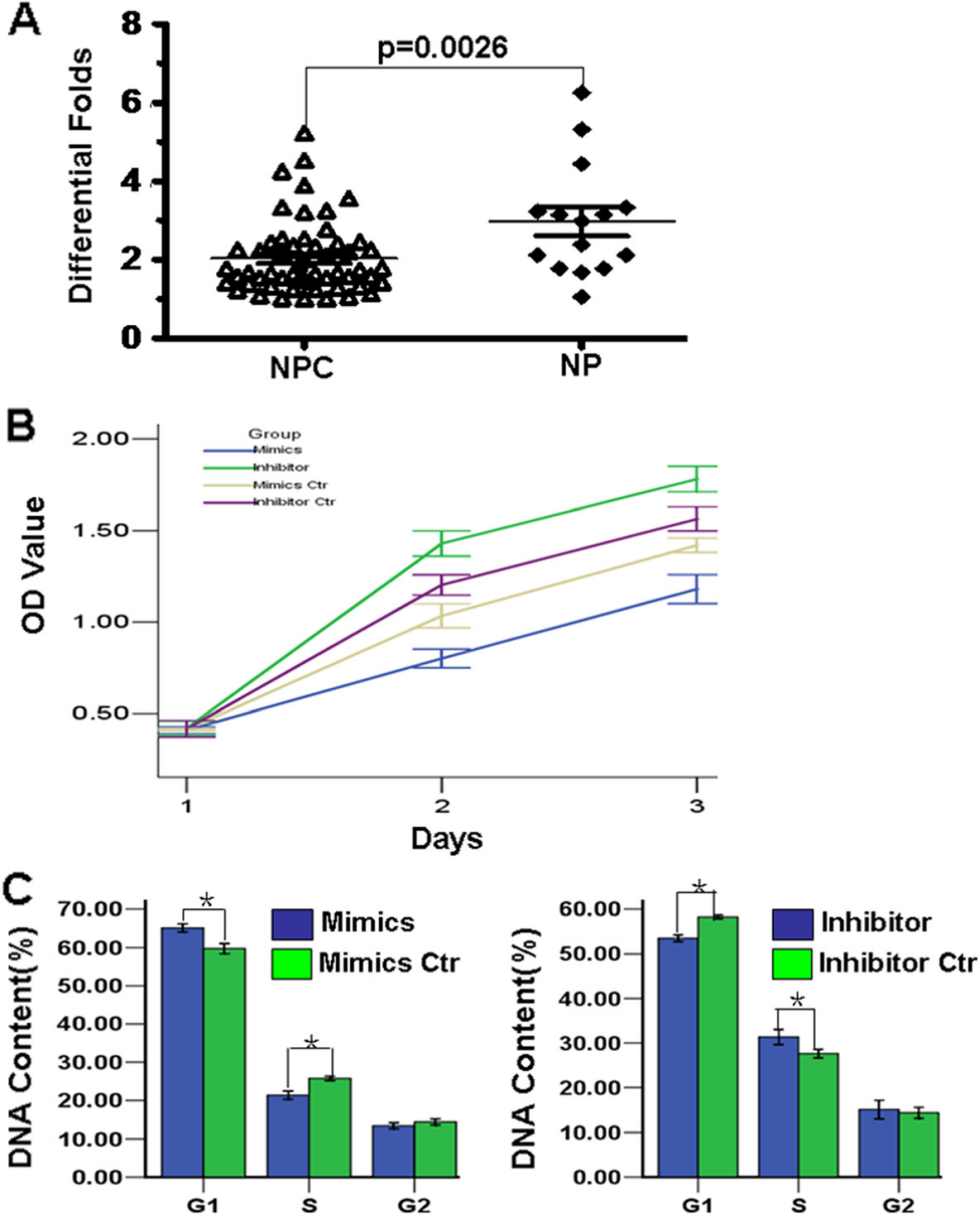
**Let-7c is downregulated and suppressed cell growth and cell cycle progression. A**. Compared to NP tissues, let-7c expression was reduced in NPC tissues. **B**. Let-7c mimics or its inhibitor respectively suppressed or promoted cell growth. **C**. Let-7c mimics or its inhibitor respectively suppressed or promoted G1 to S phase cell cycle progression.

Downregulated expression of let-7c has been confirmed in some tumors [15,17]. However, its expression pattern has still not been examined in NPC. In this study, we examined the differential expression of let-7c between 56 fresh NPC tissues and 15 fresh nasopharyngeal tissues but not formalin-fixed, paraffin-embedded (FFPE) tissues. The results showed that let-7c was significantly reduced in NPC tissues compared to nasopharyngeal tissues (Figure 3A), suggesting that let-7c might play a suppressive role in NPC pathogenesis.

### Let-7c suppresses cell proliferation and cell cycle progression in NPC

To investigate the effect of let-7c, we introduced let-7c mimics or its inhibitor respectively into NPC 5-8F and 6-10B cells. Compared with their negative controls, we found that let-7c mimics or its inhibitor respectively inhibited or promoted cell growth and cell cycle progression in NPC cells by MTT and Cytometry assays (Figure 3B,C). Our results demonstrated that let-7c is a potential tumor suppressor in NPC.

### Let-7c modulates p15/p16/CDK4/E2F1 in NPC

**Figure 4:**
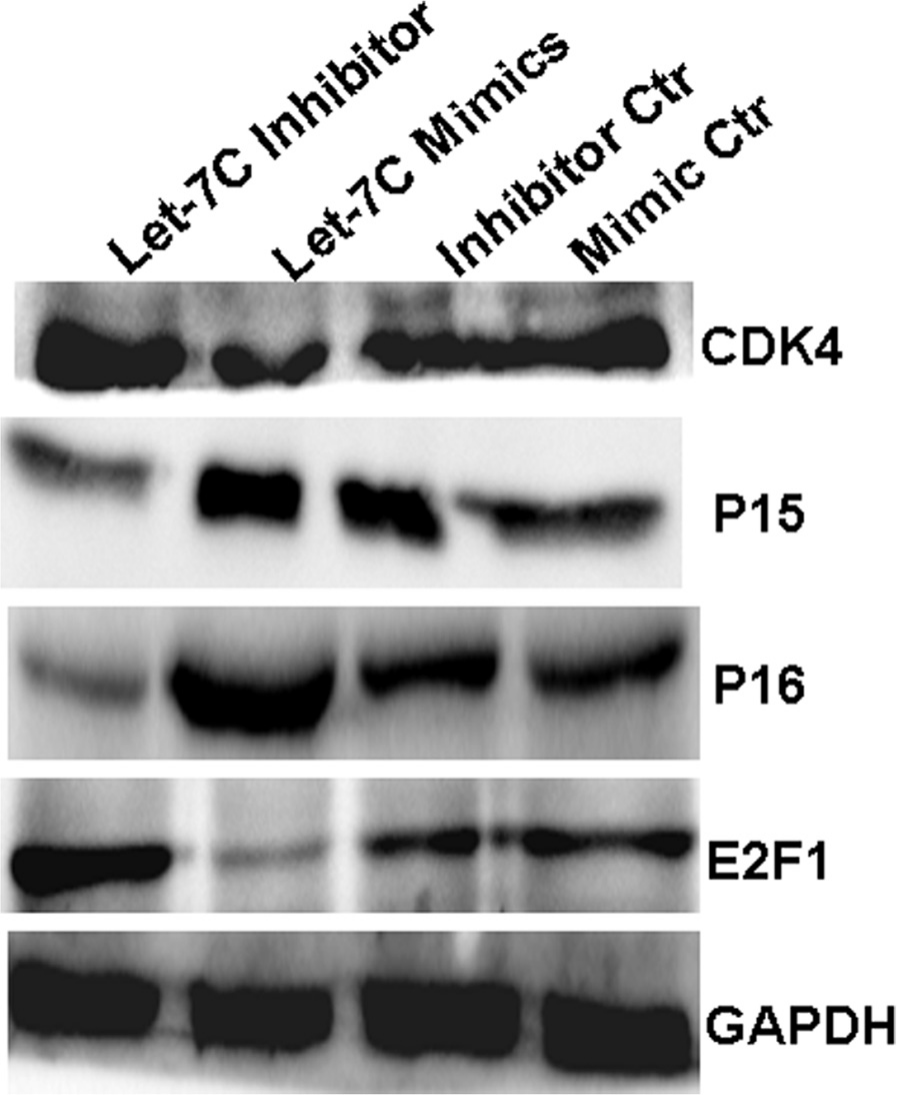
**Let-7c modulates cell cycle signaling.** Let-7c mimics elevated p15 and p16 expression and decreased and the expression of CDK4 and E2F1 while its inhibitor had the opposite effect.

In a previous investigation, let-7c was reported to directly target C-Myc, thereby suppressing cell growth [18]. In this study, we found that let-7c mimics elevated p15 and p16 expression and decreased the expression of CDK4 and E2F1 in NPC 5-8F cells. Contrary to the specific effect caused by the introduction of let-7c mimics, we observed that the expression of p15 and p16 was inhibited and the expression of CDK4 and E2F1 was enhanced by using let-7c inhibitor in NPC 6-10B cells (Figure 4).

### Overexpression of CDK4 is associated with NPC progression and poor prognosis

**Figure 5:**
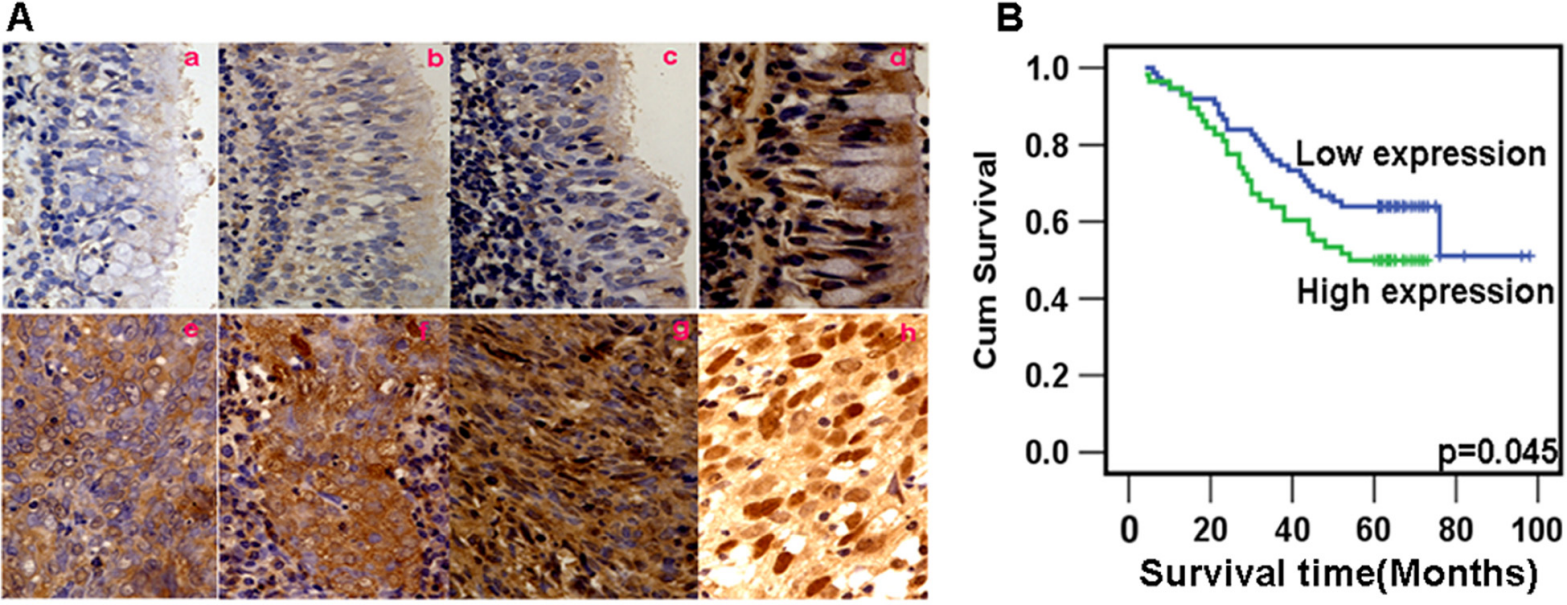
**Overexpressed CDK4 and non-treatment of radiotherapy are associated with poor prognosis of NPC. A**. CDK4 was expressed in NPC and NP tissues. **a**,**b**, and **c**: Weak cytoplasmic expression of CDK4 in NP tissues. **d**: High cytoplasmic expression of CDK4 in NP tissue. **e**: High cytoplasmic expression of CDK4 in NPC tissues. **f** and **g**: High cytoplasmic expression but low nuclear expression of CDK4 in NPC tissues. **h**: High cytoplasmic and nuclear coexpression of CDK4 in NPC tissues. **B**. Overexpressed CDK4 was associated with poor prognosis of NPC.

Overexpression of CDK4 has been reported in NPC, however, the correlation of CDK4 expression with clinical features and prognosis of NPC has not been documented. In this study, specific CDK4 protein staining was found in the cytoplasm and nucleus of normal and malignant nasopharyngeal tissues by immunohistochemistry (Figure 5A). CDK4 protein was highly expressed in NPCs compared to NP tissues (p = 0.001) (Table 1). Furthermore, we observed that overexpressed CDK4 was positively associated with tumor clinical stage (I-II vs. III-IV) (p = 0.047), but not correlated with patient's age, sex, smoking, lymph node metastasis (N classification), tumor size (T classification), and distant metastasis (M classification) in NPC (Table 2). Further, we investigated the prognostic value of CDK4 expression for NPC patients and observed that the level of CDK4 protein expression had significantly correlated with overall survival (Figure 1F). Patients with higher levels of CDK4 expression had poorer survival rates than those with lower levels of CDK4 expression (Figure 5B). Univariate analyses showed that radiotherapy, T, N, M classifications and clinical stages were also significantly correlated with patients’ survival (P = 0.002, P<0.001, P = 0.002, P<0.001, and P<0.001 respectively). NPC patients with the treatment of radiotherapy had markedly better overall survival rates than those without the treatment of radiotherapy (p = 0.001).We performed multivariate analysis of CDK4 protein expression levels adjusted for all factors and found that the level of CDK4 expression indicated a tendency as an independent prognostic factor for NPC (P = 0.087) (Table 3).

**Table 2:** Correlation between the clinicopathologic characteristics and expression of CDK4 protein in NPC

**Characteristics**	**n**	**CDK4 (%)**		***P***
		**Low expression**	**High expression**	
Gender				
Male	92	41	51	
Female	41	17	24	0.850
Age(y)				
≥50	67	29	38	
<66	66	29	37	1.000
Smoking				
No	111	50	61	
Yes	22	8	14	0.490
T classification				
T_1_-T_2_	99	41	58	
T_3_-T_4_	34	17	17	0.426
N classification				
N_0_-N_1_	74	30	44	
N_2_-N_3_	59	28	31	0.483
Distant metastasis				
Yes	10	3	7	
No	123	55	68	0.512
TNM Clinical stage				
I~II	50	16	34	
III~IV	83	42	41	0.047

**Table 3:** Summary of univariate and multivariate Cox regression analysis of overall survival duration

**Parameter**	**Univariate analysis**			**Multivariate analysis**		
	***P***	**HR**	**95% CI**	***P***	**HR**	**95% CI**
Gender						
Male vs. female	0.583	1.176	0.660-2.097	0.930	1.027	0.560-1.885
Age						
≥50vs. <50 years	0.352	1.281	0.760-2.159	0.933	0.977	0.568-1.680
Family tumor history						
Yes vs. No	0.970	1.022	0.319-3.273	0.917	0.940	0.289-3.052
Smoking						
Yes vs. No	0.979	1.010	0.495-2.060	0.452	1.340	0.625-2.876
Chemotherapy						
Yes vs. No	0.585	1.220	0.598-2.489	0.709	1.171	0.512-2.679
Radiotherapy						
Yes vs. No	0.002	0.308	0.145-0.654	0.009	0.280	0.108-0.727
T classification						
T_1_-T_2_ vs. T_3_-T_4_	0.000	2.611	1.533-4.446			
N classification						
N_0_-N1 vs. N_2--_N_3_	0.002	2.317	1.367-3.927			
M classification						
M_0_ vs. M_1_	0.000	7.280	3.519-15.061			
Clinical stage						
I-II vs. III-IV	0.000	5.222	2.467-11.054	0.000	4.527	2.041-10.042
Expression of CDK4						
High expression vs. low expression	0.099	1.554	0.920-2.626	0.087	1.671	0.928-3.008

## Discussion

Dysfunction of cell cycle signaling is one of main features in NP carcinogenesis [10,19]. CDK4, a member of the cyclin-dependent kinase family, is a key factor of cell cycle signal affecting cell cycle progression and its overexpression has been described in many tumors, including NPC. However, CDK4-mediated molecular mechanisms linked to the initiation and development of NPC are not completely understood.

In previous studies, CDK4 had been shown to promote cell proliferation by driving cell cycle progression [20-26]. To understand the biological functions of CDK4 in NPC, we first constructed NPC cells with stable suppression of CDK4 protein. We observed that knocking down CDK4 inhibited cell growth and G1 to S phase cell cycle progression. Our results are similar with these previous reports of CDK4 function in other tumors. CDK4 mediating miRNA expression to modulate the pathogenesis of NPC was not been reported. In this study, we examined the expression of tumor-suppressive miRNA let-7c by qPCR in NPC cells after knockdown of CDK4. Interestingly, let-7c expression was significantly upregulated after suppressing CDK4 expression which strongly suggested that CDK4 regulated let-7c expression in NPC. In previous reports, CDK4 was observed to be a nuclear expressed protein that forms a complex with CCND1, CDK6, and p21, and modulates the expression of pRB activating transcription factor E2F1 [27]. In further analyses, predicted binding sites of transcription factor E2F1 were found in the putative let-7c promoter, suggesting that E2F1 might modulate let-7c expression. Therefore, we suspected that knocking down CDK4 stimulated let-7c expression might be attributed to the suppression of E2F1. Consistent with this expectation, we discovered that knocking down CDK4 elevated tumor suppressor p21 and inactivated oncocogenic factors CCND1, CDK6, and E2F1 in NPC cells. Subsequently, we observed that inhibiting E2F1 by specific siRNA increased the expression of let-7c in NPC cells.

Let-7c has been identified as a tumor suppressor in some tumors [15,16]. However, its roles in NPC have not been yet reported. In this study, we found that let-7c was significantly decreased in NPC tissues compared to nasopharyngeal tissues. Further, we observed that let-7c inhibited cell growth, migration, and invasion of NPC cells. These results suggested that let-7c functions as a potential tumor suppressor in NPC. In a previous study, let-7c was reported to directly target C-Myc-mediated CDK4 suppression blocking cell growth in some tumors. In this investigation, we observed that p15 and p16 [28-31], two tumor suppressors that are upstream regulators of CDK4 [32] were positively modulated by let-7c, whereas CDK4 and E2F1 were negatively regulated by let-7c in NPC cells. These results suggested that let-7c suppressed cell growth through p15/p16/CDK4/E2F1 signaling in NPC. More interestingly, a positive feedback loop of CDK4-E2F1-let-7c was observed, which was similar to our previous report for CTGF-C-Jun/C-Myc-miR-18b in NPC which promoted NPC pathogenesis [13].

Increased expression of CDK4 has been reported in NPC [12]. However, the correlation of CDK4 expression with clinical features and prognosis of NPC has not been documented. In this study, we observed that CDK4 was mainly coexpressed in the nucleus and cytoplasm in lung cancer and normal lung tissues. Furthermore, we found that total protein levels of CDK4 were overexpressed in NPC tissues compared to normal NP tissues. These results were analogous to our previous reports in lung cancer [12], suggesting that CDK4 participates in the pathogenesis of NPC.

CDK4 expression patterns had been reported to be associated with clinical pathology parameters in some tumors including lung cancer, osteosarcomas, colorectal cancer, and chondrosarcomas [12,33-35]. In this study, we observed that overexpressed CDK4 was positively associated with clinical stage, but not correlated with patient's age, sex, smoking, or T classification, N classification, and M classification in NPC. Further, we observed that the level of CDK4 protein expression was significantly correlated with the overall survival of NPC patients. Patients with higher levels of CDK4 expression had poorer survival rates than those with lower levels of CDK4 expression. NPC is highly sensitive to radiotherapy. In this study, we also observed that NPC patients with the treatment of radiotherapy had markedly better overall survival rates than those without the treatment of radiotherapy, which indicated the significance of radiotherapy for NPC patients. Finally, we found that although CDK expression was not significantly associated with overall survival of NPC patients according to univariate analyses, it seemed that its overexpression showed a tendency as an independent prognostic factor for NPC patients regardless of its patients' disease status based on multivariate analyses.

## Conclusions

In summary, our study demonstrated that knocking down CDK4 induced the activation of let-7c by modulating the p15/p16/CDK4/E2F1 pathway, which in turn suppressed cell proliferation by controlling p21/CCND1/CDK4/E2F1 signaling. Furthermore, we observed that overexpressed CDK4 is an unfavorable factor which promotes progression and poor prognosis of NPC.

## Competing interests

All the authors declare that they have no competing interests.

## Authors’ contributions

ZL, ABW, XBL, CC, YC, QYW, SNH, and YJZ performed the study and coordination and assisted in editing of manuscript. ZL, and CC collected tissue samples. WYF, ABW and YJZ designed this study and wrote this paper. All authors read and approved the final manuscript.

## Pre-publication history

The pre-publication history for this paper can be accessed here:

http://www.biomedcentral.com/1471-2407/14/274/prepub
